# Development of national system performance metrics for tissue donation, production, and distribution activity

**DOI:** 10.1007/s10561-017-9637-2

**Published:** 2017-07-01

**Authors:** Brenda Weiss, Mazen Dakkak, Gary Rockl, Balram Sukhu, Jim Mohr, Kyle Maru, on behalf of the Eye and Tissue Data Committee

**Affiliations:** 1 Ophthalmology Clinic, Misericordia Eye Bank, Misericordia Health Centre, 99 Cornish Ave, Winnipeg, MB Canada; 2 Héma-Québec, 1070, avenue des Sciences-de-la-Vie, Quebec City, QC Canada; 3 Southern Alberta Tissue Program, Foothills Medical Center, McCaig Tower Rm 4510, 1403 29th St. NW, Calgary, AB Canada; 4 Mount Sinai Hospital, 600 University Ave, Toronto, ON Canada; 5 Canadian Blood Services, 270 John Savage Ave, Dartmouth, NS Canada; 6 Canadian Blood Services, 2713 Lancaster Rd, Ottawa, ON Canada

**Keywords:** Canada, Statistics, Data, Eye, Tissue, Donation, Transplantation

## Abstract

Canada’s federal, provincial, and territorial governments gave Canadian Blood Services a mandate for organ and tissue donation and transplantation, including system performance, data and analytics. In 2012 Canadian Blood Services facilitated an eye and tissue banking workshop focused on standardized specifications and practices. At the workshop, the Canadian tissue community directed Canadian Blood Services to facilitate the development and implementation of a national data stream and analytics. Prior to this no national data was prospectively collected or collated on tissue donation, production or distribution activity. An eye and tissue data committee was formed with representation from eye and tissue banks in all Canadian jurisdictions. A minimum data set, standardized definitions, a data submission form and a quality assurance process was developed. Training was provided to data personal identified by each eye and tissue bank. Data collection was initiated January 1, 2013; with quarterly data submitted to Canadian Blood Services via excel spreadsheet. Data was submitted by sixteen Canadian eye and tissue banks, located in eight of Canada’s thirteen provinces and territories, representing a census of activity. Annual data reports, with trend analysis, are generated and distributed to the tissue community to inform operational strategy and system performance improvement. This report provides an overview of the data process and provides visibility to the Canadian tissue donation, production and distribution activities for 3 years; January 1, 2013 to December 31, 2015.

## Introduction

Canadian Blood Services manages the national supply of blood, blood products, stem cells, a cord blood bank and related services for all the provinces and territories (excluding Quebec). It also leads an integrated, interprovincial system for organ donation and transplantation for all of Canada. In 2008 Canada’s federal, provincial, and territorial governments gave Canadian Blood Services a mandate for organ and tissue donation and transplantation, including system performance, data and analytics. In 2012 Canadian Blood Services facilitated an eye and tissue banking workshop focused on standardized specifications and practices. At the workshop, the Canadian tissue community directed Canadian Blood Services to facilitate the development and implementation of a national data stream and analytics. Prior to this no national data was collected or collated on tissue donation, production or distribution activity.

Canadian eye and tissue banks in collaboration with Canadian Blood Services support the collection and analysis of national data on tissue donation, allograft production and distribution activity. An Eye and Tissue Data Committee (ETDC) was established in 2012, co-chaired by a member of the tissue community and a member of Canadian Blood Services to provide oversight for the collection, maintenance, collation and release of activity data (Appendix A).

Oversight and recommendations to the larger committee in relation to data elements, data definitions (Appendix B), data collection, data submission, quality assurance and training, collation, analysis and release and publication are provided by two working groups derived from ETDC members, The Minimal Data Set Working Group and the Publication Working Group. Canadian Blood Services is the repository for the collected data and supports data management, analytics, and provides secretariat support for the ETDC’s analysis and publication of data.

**Fig. 1:**
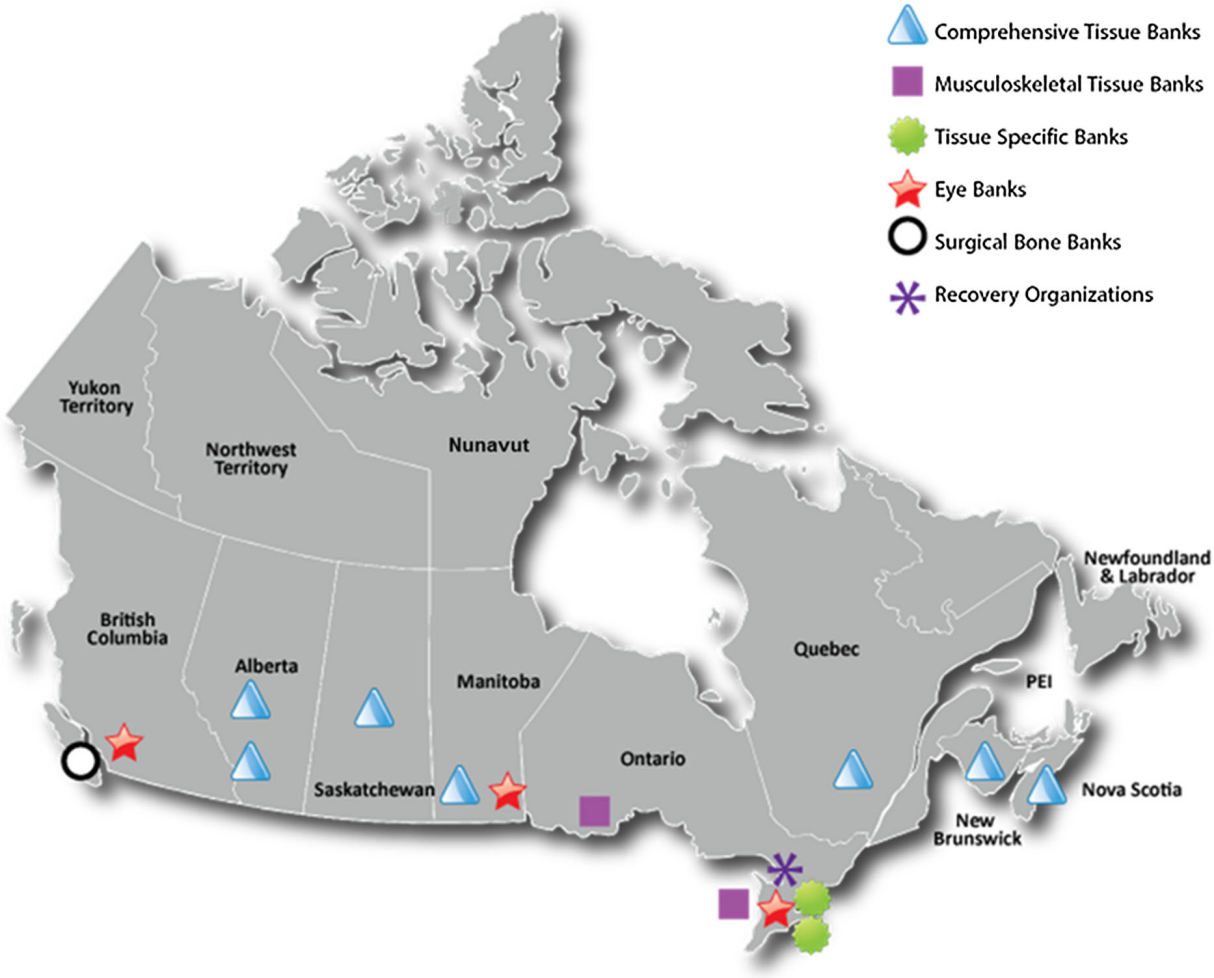
Canadian Eye and Tissue Banks

Prospective data collection was initiated in 2012 from all eye and tissue banks operating in Canada (Appendix C). Data was submitted by the Canadian eye and tissue banks operating in eight out of the thirteen provinces and territories (Fig. 1). This represents a census of eye and tissue banking activity in Canada. The only data set which is not a census of Canadian activity is *approach and consent rate*; the results presented are based on the thirteen programs which submitted data on this metric in order to provide visibility to the consent rate within those programs.

It is recognized that many hospitals import allografts directly from the United States; and more specifically demineralized bone products, acellular dermal matrix and other advance highly processed products not currently produced by Canadian Banks. Data on allografts imported by Canadian hospitals directly from United States banks is not readily available; this analysis represents data on the donation, production and distribution activity of Canadian eye and tissue banks.

The results presented here report on Canadian eye and tissue banking donation, production, and distribution statistics for Canadian eye and tissue banks for January 1 to December 31, 2015 as well as Canadian system activity for 2013, 2014 and 2015.

**Fig. 2:**
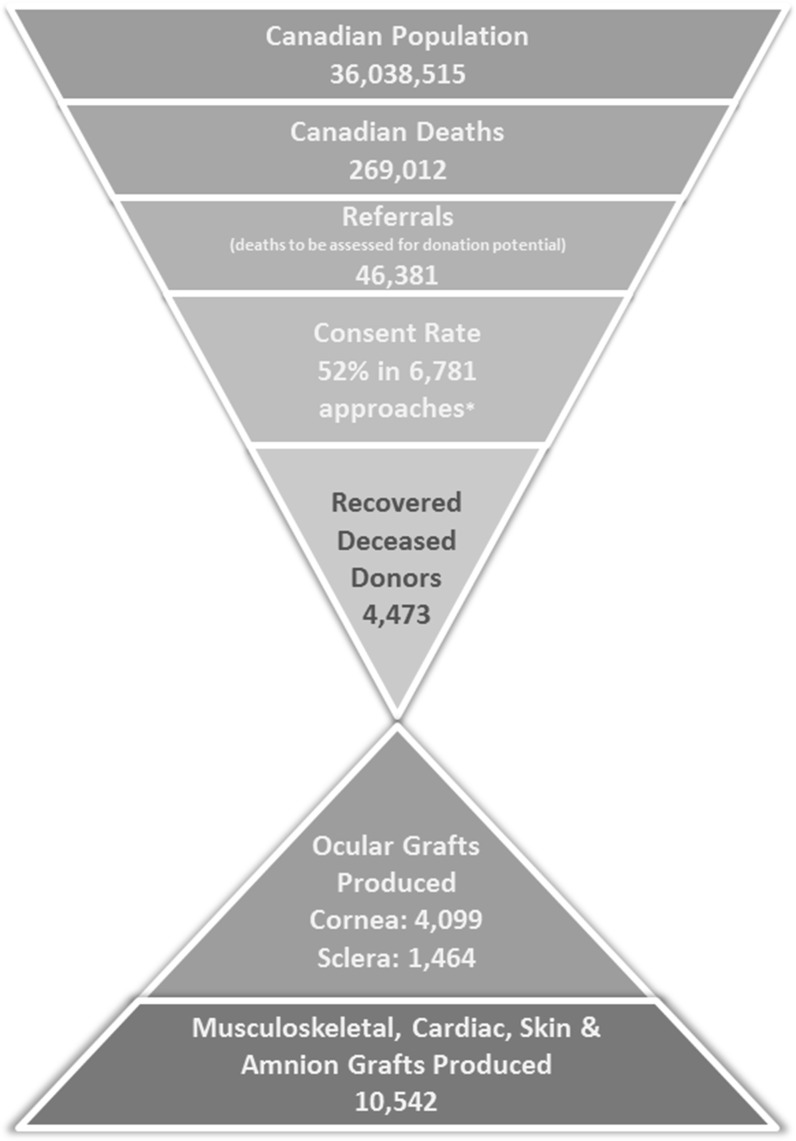
Canadian overview of tissue donation and transplantation, 2015. [Figure adapted from potential organ donor population and transplant outcomes 2015 (Australian Government 2016: 27). Canadian Population reflects updated postcensal estimate for January 1, 2016 (Statistics Canada 2016a). Canadian Deaths reflect preliminary totals for July 1, 2015 to June 30 2016 (Statistics Canada 2016b)]

Figure 2 illustrates the total number of deceased donors from which ocular and/or tissue grafts were recovered in 2015 relative to the overall consent rate and number of approaches for consent, the number of deaths referred for donation, the number of deaths in Canada, and the Canadian population, as well as the number of grafts produced by graft type (not to scale).

## Canadian eye and tissue banking deceased donation activity

### Total donor referrals

A total of 46,381 deaths were identified and referred for initial screening/consideration of tissue donation in 2015, an increase of 2.7% over 2014 referrals. (n = 45, 154).

**Fig. 3:**
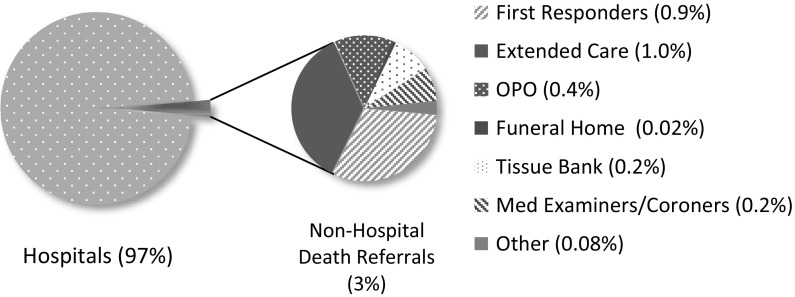
Death referrals by source, 2015 (n = 46,381)

Figure 3 presents the proportion of death referrals nationally in 2015 by the source of the referral. Because hospital referrals comprise 97% of all referrals nationally, non-hospital referrals are distinguished.

### Consent rate

In 2015, 13 programs were able to provide data on 6781 approaches for deceased tissue donation. 89% of approaches were made by tissue bank personnel, the remainder by front line health care professionals. A consent rate of 52% was identified. Data is not currently available on consent rate by tissue type.

**Fig. 4:**
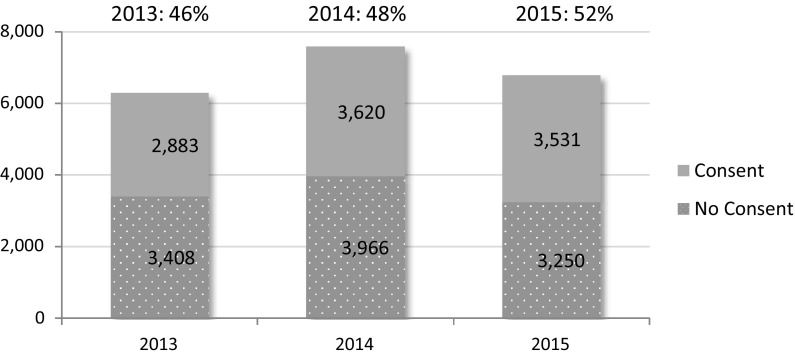
Consent rate for tissue donation by year

Figure 4 provides the overall consent rate based on approaches recorded by year (2013–2015), showing the number of approaches resulting in an obtained consent relative to the number of approaches that did not result in consent being obtained. Although a greater number of consents were recorded for 2014, the consent rate in 2015 was marginally higher.

### Deceased donor breakdown and analysis

In 2015 there were 4473 deceased tissue donors in Canada. The vast majority of these donors (87% n = 3883) were ocular only donors. The total number of deceased donors has remained relatively stable over the last 3 years; however there has been a 24% (n = 182) decrease in the number of donors where musculoskeletal, skin or cardiac tissue was recovered since 2013. The results in 2015 evidence no change in ocular-only donors relative to 2014, making up 86.8% (n = 3883) and 86.1% (n = 3883) of donors, respectively. Donors who made both ocular and tissue donations represented 9.1% (n = 409) of donors overall in 2015, which is also consistent with their representation among donors in 2014 (8.1% n = 365) and 2013 (12.2% n = 516).

**Fig. 5:**
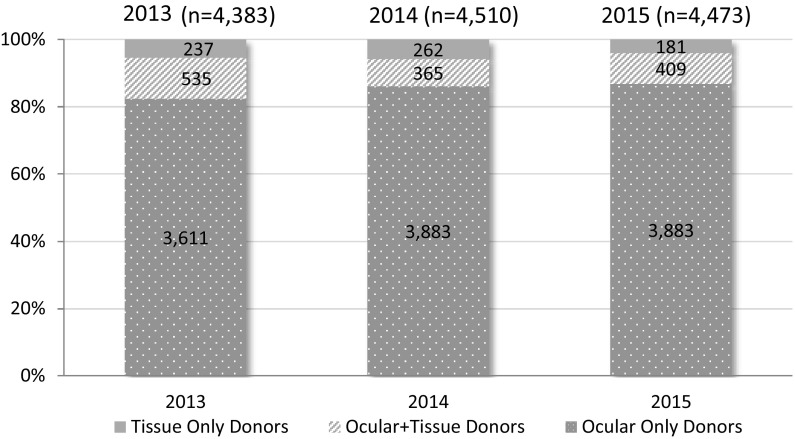
Deceased donors by tissue type recovered and year

Figure 5 presents the counts and relative proportions of deceased donors by year (2013–2015) based on the type(s) of tissues recovered.

## Canadian eye and tissue banking living donation activity

### Living donors: surgical bone

In 2015, five programs reported recovering bone from living donors; recovering femoral heads during total hip replacement surgery. There has been a 22% (n = 151) decrease in surgical bone donation between 2013 and 2015. There has been an 18% decrease in 2015 (n = 120) from 2014 levels. The number of surgical bone grafts distributed for transplant continues to decrease in line with the decrease in recoveries.

**Fig. 6:**
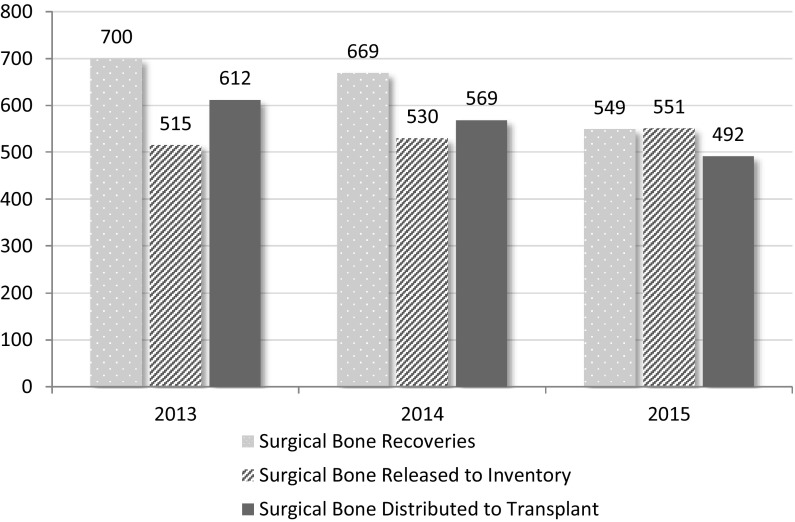
Surgical bone graft recovery, release to inventory, and distribution for transplant by year

Figure 6 shows the number of surgical bone grafts recovered, released, and distributed for transplant by year (2013–2015). The number distributed may exceed the number released since grafts are not necessarily distributed in the same year that they are released.

### Living donors: amnion

In 2013, three programs reported recovering amnion from 9 living donors, producing 203 grafts, and distributing 153 grafts for transplant. In 2014 and 2015 there were four programs recovering amnion; in 2014, these programs produced 598 grafts from 12 living donors and distributed 502 grafts for transplant, while in 2015 these programs distributed 271 grafts from 8 living donors with 384 grafts distributed to transplantation that year. Although the number of grafts produced per donor has been consistent from 2014 to 2015 at 49.8 and 48.0 grafts per donor respectively, this is more than double the per donor amnion production rate reported in 2013 (22.6 amnion grafts per donor).

**Fig. 7:**
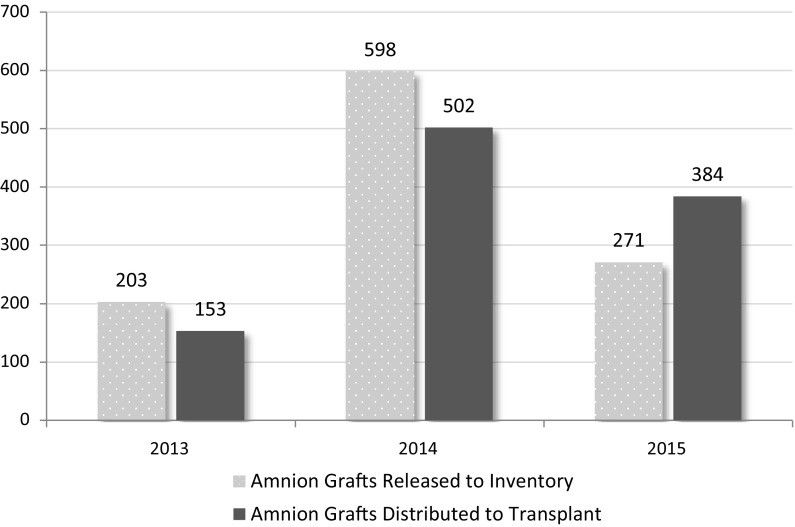
Amnion grafts released to inventory and distributed to transplant by year

Figure 7 shows the number of amnion grafts released and distributed for transplant by year (2013–2015). The number distributed may exceed the number released since grafts are not necessarily distributed in the same year that they are released.

## Canadian eye and tissue production and distribution activity

### Corneas distributed for transplant

In 2015, Canadian eye banks produced/released 4099 corneas for transplant, a 5.6% (n = 245) decrease from the 2014 production activity of 4344 corneas. Of those 3097 were distributed for cornea transplant (keratoplasty) as compared with 3259 in 2014; a decrease of 5.0% (n = 162). An additional 263 corneas were utilized in non-keratoplasty procedures including glaucoma shunt patch. A limitation in the data is corneas where the final use was detailed as “unknown” which included an additional 64 corneas in 2015 a significant improvement over the 632 unknown in 2014.

51% (n = 1586) of all cornea transplants performed in Canada in 2015 were Endothelial Keratoplasty (EK) as compared to 49% (n = 1600) in 2014.

In 2015, four Canadian eye banks provided DSAEK processing service. In remaining regions the processing is completed by the surgeon in the operating room. No Canadian eye banks provide DMEK; however a number are planning implementation of this service within the next 18 months.

**Fig. 8:**
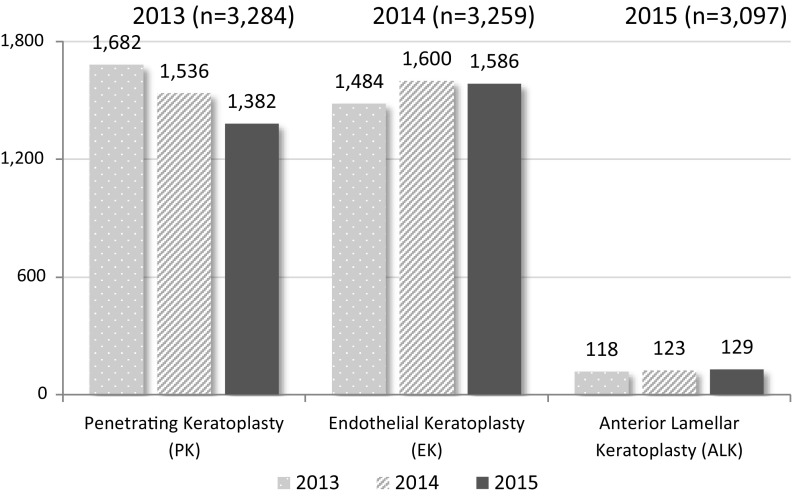
Type of keratoplasty (Cornea transplant)

Figure 8 shows the number of cornea transplants (keratoplasty) by year (2013–2015) by transplant procedure.

In endothelial keratoplasty (EK), either the eye bank prepares the corneal tissue, or the surgeon prepares the corneal tissue in the operating room, removing specific layers of the cornea. There are two common methodologies; Descemets Stripping (automated) Endothelial Keratoplasty (DSAEK) and Descemets Membrane (manual) Endothelial Keratoplasty (DMEK). The DMEK peel has been described as a more technically challenging procedure than DSAEK but also has been reported to provide better, post-transplant patient visual acuity, lower rejection rates and faster visual recovery. The demand for DMEK continues to increase; representing 24% of EK corneas in 2015 as compared to 6% in 2013. In 2015 DMEK procedures were performed by transplanting ophthalmologists.

**Fig. 9:**
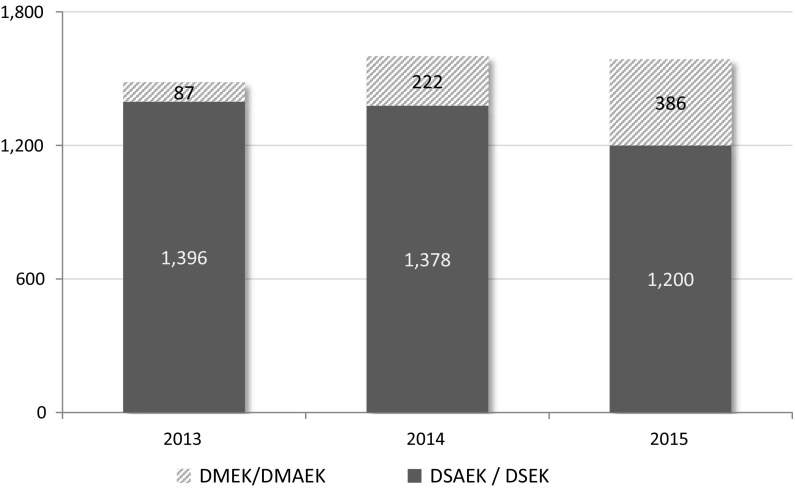
Type of endothelial keratoplasty

Figure 9 shows the number of endothelial keratoplasty (EK) procedures performed by EK methodology and year (2013–2015).

### Tissue grafts processed and released to inventory

In 2015, ten tissue banks processed and released 10,678 musculoskeletal, cardiac, skin and amnion grafts, from deceased and living donors into inventory for transplant; detailed as 42% (n = 4437) cancellous bone products, 23% (n = 2371) skin grafts, 16% (n = 1707) tendons, 9% (n = 936) structural bone, 3.9% (n = 551) surgical bone and 2% (n = 221) cardiac grafts.

There is essentially no change in total production from 2014 (n = 10,837) but a substantial reduction from the 2013 (n = 12,045) production; a 11% decrease (n = 1367). In 2015 there has been a 16% increase in cancellous production (n = 622), a 20% increase in tendon production (n = 280), a 65% decrease in small structural graft production (n = 160), a 55% decrease in amnion production (n = 327) and a 14.5% decrease in skin graft production (n = 403).

**Fig. 10:**
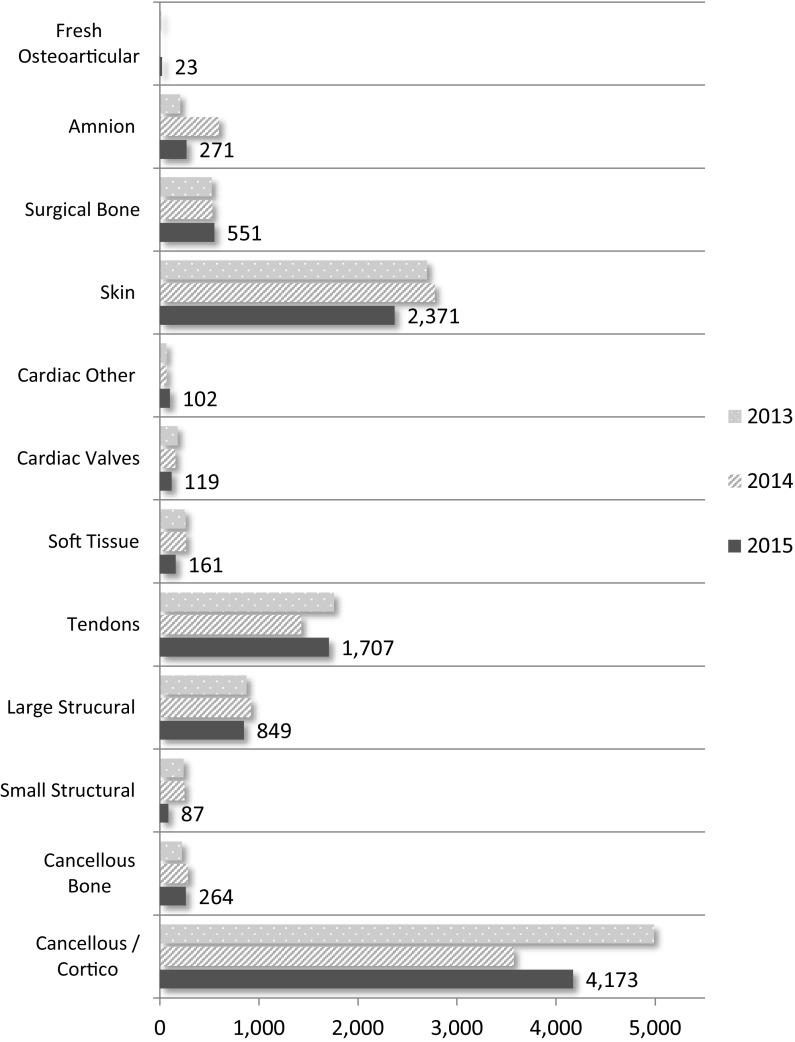
Number of grafts processed and released to inventory

Figure 10 shows the number of tissue grafts processed and released to inventory by graft type and year (2013–2015). Numerical values are provided for 2015 results.

### Tissue grafts distributed to transplant

In 2015, eleven tissue banks distributed 12,119 processed grafts for transplantation; essentially unchanged from the 2014 (n = 11,740) and 2013 (n = 12,605) distribution. There has been a 37% (n511) increase in the distribution of tendons since 2013 and a 27% decrease in amnion distribution since 2014 (n = 135). While ten banks produce allografts an eleventh has a relationship with American processors who produce allografts from donors recovered by that bank and return them for distribution. Canadian banks distribute processed grafts within their own provinces and to other provinces. There is no distribution outside of Canada, with the exception of a small number of corneas which were deemed unacceptable for use in Canada but accepted overseas.

**Fig. 11:**
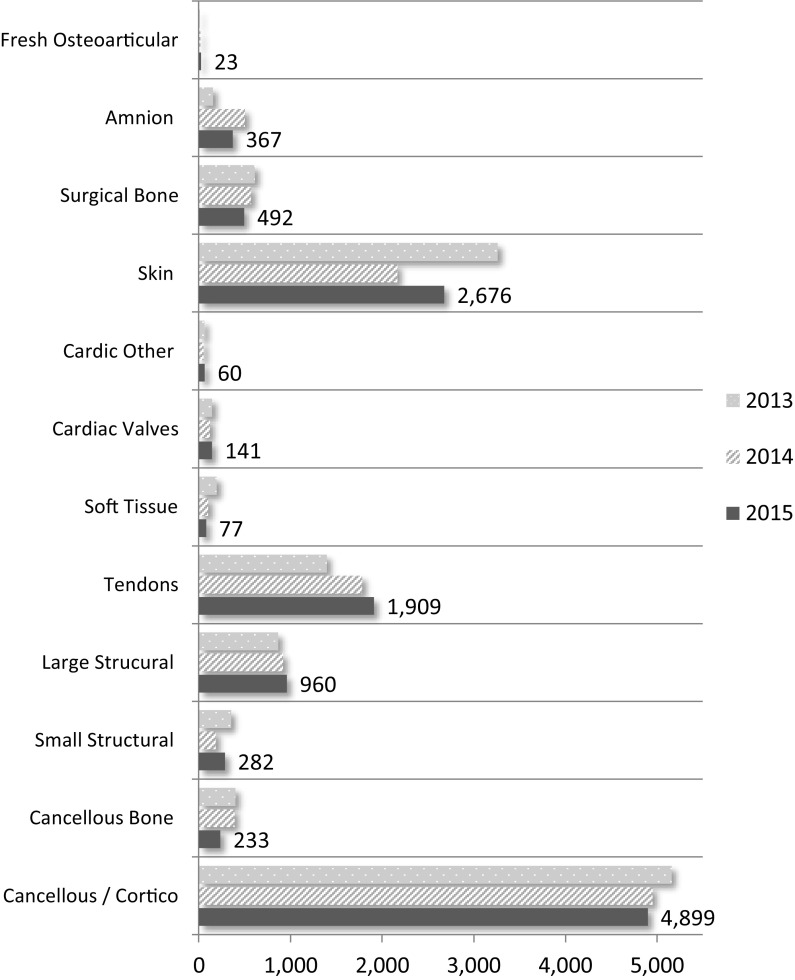
Number of grafts distributed to transplant

Figure 11 shows the number of tissue grafts distributed to transplant by graft type and year (2013–2015). Numerical values are provided for 2015 results.

### Comparative analysis Canadian eye and tissue banking activity

See Table 1.

**Table 1:** Overview of Canadian eye and tissue banking activity

Total Canadian activity	2013	2014	2015	% Change 2014–2015
Deaths referred to be assessed for donation potential	41,594	45,154	46,381	2.7
Total deceased donors recovered	4383	4510	4473	−0.8
Donors where ocular tissue was recovered	4146	4248	4292	+1.0
Deceased donors where bone, cardiac and or skin was retrieved	772	627	590	−5.9
Surgical bone donors	700	669	549	−17.9
Corneas produced and released for transplant	4004	4344	4099	−5.6
Corneas transplanted—keratoplasty	3284	3259	3097	−5.0
Cornea yield: number of corneas processed/released per ocular donor	0.97	1.02	0.96	−5.9
Total bone, skin and cardiac grafts processed and released into inventory in deceased donors	11,297	9709	9856	+1.5
Total non-ocular grafts distributed to transplantation (bone, skin, cardiac, surgical bone, amnion grafts)	12,605	11,740	12,119	+3.2
Total all eye and tissue grafts processed and released to inventory (deceased and living donors: ocular, bone, skin, cardiac, surgical bone, amnion)	17,602	16,570	16,241	−2.0

## Discussion

Canadian eye and tissue banks operate within publically funded provincial health care systems. The majority of banks are located, and funded, within hospital environments. In Quebec the tissue program has been incorporated within the provincial blood agency, Héma-Québec. There are no private for-profit tissue banks in Canada.

Eye and tissue bank production is, for the most part, focused to support demand within provincial regions. A small number of tissue banks have developed a business model which includes distribution outside their regions using a cost recovery model. Eye banks have only recently begun to recover costs for corneas distributed between provinces.

Canadian tissue banks have not developed the capability to produce advanced tissue products such as demineralized bone, machined bone grafts and acellular dermal matrix. Market analysis indicates Canadian hospitals continue to import, from the United States, approximately 20,000 advanced surgical and 30,000 dental tissue products annually at a cost of approximately $50 million. Tissue banks indicate a lack of human resources, research and development and capital funding and the constraints of operating within a hospital environment hamper the development of advanced processing capability. In addition as there is ready access to advanced grafts from US processors there is no pressure from clinicians for Canadian banks to provide these products.

In recent years a number of provinces have imported corneas from the United States to supplement their local production and address waiting lists for transplantation. The Eye and Tissue Data Committee has revised data collection to quantify the importation of corneas from the United States. This data will be available in future years for analysis.

In 2015 Canadian eye and tissue banks received 46,381 referrals for potential tissue donors. Referral processes vary between provinces. The majority of Canadian provinces have, or are advancing mandatory referral, where all deaths with the potential for tissue donation are referred to a donation organization or tissue bank.

The consent process varies between provinces. The majority of consents are obtained by trained donation organization or eye and tissue bank staff. However, in a number of jurisdictions front line staff obtains consent.

Of those approached for consent 52% consent to tissue donation. Tissue was recovered from 4473 deceased donors and 557 living donors resulting in the production of 16,241 grafts released into inventory for distribution; 5563 ocular (cornea and sclera) and 10,678 tissue (bone, skin, cardiac, surgical bone and amnion). This represents a 2% production decrease from 2014, mainly from a decrease in realised ocular tissue; and a total allograft production decrease of 7.7% from 2013.

Overall a 0.8% decrease in deceased donor’s recovered between 2014 and 2015 and a decrease in living tissue donation by 18% between 2014 and 2015.

There was a 1.0% increase in the number of donors where ocular tissue was recovered yet a 5.6% decrease in corneas produced and released to inventory and a 5.0% decrease in corneal transplants (keratoplasty) between 2014 and 2015. There was a 5.9% decrease in the yield of cornea grafts (# corneas released for transplant per donor) decreasing from 1.02 to 0.96 per donor between 2014 and 2015.

Results indicate that in 2015, 51% of all cornea transplants (keratoplasty) performed in Canada were endothelial keratoplasty; requiring post recovery processing of the cornea prior to transplantation. Demand for Descemet Membrane Endothelial Keratoplasty (DMEK) increased significantly in both 2014 and 2015 and now accounts for 24% of all endothelial keratoplasties.

In 2015 there was a 5.9% decrease in the number of deceased donors where musculoskeletal, skin and or cardiac tissue was recovered and a decrease of 23.6% from 2013.

There was a 1.5% increase from 2014 to 2015 in the number of bone, cardiac and skin grafts processed and released to inventory from deceased donors despite a 5.9% decrease in donors. The resultant increase in released inventory was mirrored in distribution for transplant by an increase of 3.2% (11,740 to 12,119 grafts) of non-ocular grafts; musculoskeletal, skin, cardiac, surgical bone and amnion from 2014 to 2015. Amnion production dropped 55% in 2015; from 598 to 271 grafts.

Market analysis and demographics of an aging population project continuing growth in demand for tissue grafts. Yet, in an environment of increasing demand, results indicate stagnant and potentially decreased donation and production activity within Canadian eye and tissue programs.

Annual public polling data demonstrates awareness of organ and tissue donation continues to increase, approval for organ and tissue donation consistently exceeds 90% and in those who support tissue donation the vast majority indicate they would donate all of their tissues.

The lack of growth and potential decline in tissue donation activity does not appear to be related to public support. Eye and tissue banks identify a number of factors they believe have adversely affected donation activity.

The lack of mature donation registries is a significant barrier to successful consent discussions; programs have greater success in consent discussions when the donor’s wishes have been documented in a registry and can be shared with families. Provincial donor registries vary significantly in the percentage of the population registered from <5 to 50%. A number of provinces have no donor registration system at all and therefore access to registered consent documentation is inconsistent.

Eye and Tissue Banks identify continuing and increasing financial constraints within hospital and provincial health care budgets as a limiting factor in donation. Specifically, financial constraints have led to a lack of resources for appropriate staffing to support recovery, to support hospital development programs and to support development of more effective referral systems. While these challenges vary in severity between jurisdictions banks felt the cumulative effect is limiting growth in donation.

This prospective data collection provides Canadian jurisdictions with insight into tissue donation activity as well as to the Canadian production and distribution of ocular and tissue grafts. Data analysis provides valued information documenting changes in system activity between 2013, 2014 and 2015, and insight into the current tissue environment. The Eye and Tissue Data committee provides a forum for discussion where representatives of Canadian eye and tissue programs review activity metrics to identify trends and challenges which inform operational planning and strategic development.

## Conclusion

With the support of eye and tissue banks in Canada, and in collaboration with Canadian Blood Services, a census of Canadian tissue recovery, allograft production and distribution activity provides data to inform individual banks operational strategy as well as providing insight and trend analysis to inform national policy development. A data committee with representation of the majority of Canadian eye and tissue banks continues to evolve minimal data sets, data definitions, data processes and quality assurance and undertakes analysis to identify trends in activities to inform strategy.

Data collected has formed a baseline to begin informing national trends and individual tissue bank operations. Recent insights identified for consideration in operational planning and policy development include the identification of stagnant and perhaps decreasing activity in tissue donation, a concerning downward trend in the number corneal transplants and changes in corneal processing demands which have significant impacts to operational planning. The decrease identified in musculoskeletal, cardiac and skin donors and its correlative impact on allograft availability clearly decrease potential tissue supplies nationally. A decrease in corneal transplantation activity in an environment where jurisdictions are importing corneas from the US to supplement local production is of concern. Data demonstrates increasing demand for Descemet Membrane Endothelial Keratoplasty (DMEK) corneas. This trend has shifted the production methodology for ocular tissue requiring more technical expertise and training from both the surgeons and eye bank technicians.

Eye and tissue banking responses to identified challenges are shared amongst national members to inform new strategies to overcome common challenges.

The prospective collection and collation of national eye and tissue bank activity provides insight into the Canadian supply and demand. As data accumulate, more sophisticated trend analysis will help inform recovery and production targets and methodologies. Strategies to better align supply with demand nationwide can be developed using the collected data as a guide. The data collected also has the prospectus to inform further research in the ocular and tissue transplantation world, as a significant starting point for most research requires a broad tablet of basic data. The cooperation of the eye and tissue banking community and Canadian Blood Services in data collection has recently provided analysis which informed development of a national strategy, including production and inventory targets, for skin allografts to support mass causality events.

## Appendix A: Eye and tissue data committee membership

**Table:**

Member	Title	Program
Brenda Weiss (Chair)	Patient Care Manager Ophthalmology Clinic, Misericordia Eye Bank	Misericordia Health Centre, Winnipeg, MB
Mike Bentley	Manager, Transplant Services	Comprehensive Tissue Centre, Edmonton, AB
Mary Gatien	Director NB Organ Donor Program, Director NB Eye and Tissue Bank	New Brunswick Eye and Tissue Bank, Saint John and Moncton, NB
Mazen Dakkak	Business Development Officer	Héma-Québec, Québec City, QC
Ronn Ginther	Coordinator	Saskatchewan Transplant Program, Saskatoon, SK
Alison Halliday	Senior Technologist	Ontario Professional Firefighters’ Skin Bank, Toronto, ON
Christine Humphreys	Manager	Eye Bank of Canada (Ontario Division), Toronto, ON
Cynthia Johnston	Quality Leader	Regional Tissue Bank, Halifax, NS
Mijani Ridic	Unit Manager, Lions Eye Bank	Southern Alberta Organ and Tissue Program, Calgary, AB
Gary Rockl	Senior Tissue Specialist	Southern Alberta Tissue Program Calgary, AB
Natalie Smigielski	Clinical Specialist, Tissue	Trillium Gift of Life Network, Toronto, ON
Chris Snow	Director	Tissue Bank Manitoba, Winnipeg, MB
Balram Sukhu	Manager	Mount Sinai Allograft Technologies, Toronto, ON
Ivan Yan	Head Technologist	Eye Bank of British Columbia, Vancouver, BC

## Appendix B: Definitions

### Amniotic membrane

Amniotic membrane is the innermost layer of the placenta consisting of a thick basement membrane and an avascular stromal matrix. It is used as a graft and as a dressing to facilitate ocular surface reconstruction and to promote healing. Its’ use in plastic surgery (burns, wound care), orthopedic, dental and general surgery is increasing.

### Cancellous/cortical bone

There are two types of osseous tissue that form bones; cancellous “spongy” bone and cortical “compact” bone. Tissue banks mill/grind bone into cancellous cortical particles or powder which is used to pack bone voids in surgical repairs.

### Chipped bone

Is bone that has been processed into morsels which is used to pack bone voids in surgical repairs.

### Consent

Signed documentation of approval to proceed with donation from the donor or legal next of kin.

### Consent rate

Is the ratio of donors where consent for “tissue” donation is obtained to the number of donors or donor families approached for consent to “tissue” donation. Data is not currently available to differentiate consent rates between types of tissue.

### Deceased donor

Refers to a donor where tissue is recovered following cardiac or neurological death.

### Fresh osteoarticular

Osteoarticular refers to a bone graft that contains a joint surface; such as a knee. Fresh refers to the fact that in order to preserve viability of joint tissue the graft is not frozen or cryopreserved. These grafts are refrigerated and usually transplanted within weeks of recovery.

### Keratoplasty

Keratoplasty is a surgical procedure also known as corneal transplantation where the procedure is described as a replacement of abnormal host tissue with healthy corneal tissue from a donor. The replacement of the corneal tissue can either be partial or full depending on the severity of damage in the cornea.

### Penetrating keratoplasty

Corneal transplant with replacement of all layers of the cornea, but retaining the peripheral cornea.

### Endothelial keratoplasty (EK)

Endothelial keratoplasty is a corneal transplant procedure where only a patient’s compromised posterior layers of the cornea are removed and replaced by similar posterior corneal layers of a donor cornea. The advent of this procedure occurred in the early to mid-2000s after 50 years of performing penetrating keratoplasty transplant surgeries. EK has clearly established itself as the standard of care for patients with endothelial dysfunction. There are a number of types of EK procedures including DSAEK and DMEK. They can be performed manually (peel) or automated (microtome).

### Descemet’s stripping (automated) endothelial keratoplasty (DSAEK)

The vast majority of EK today is DSAEK where the eye bank precuts the corneal tissue, or the surgeon precuts the corneal tissue in the operating room. The prepared (cut) graft is comprised of the donor tissue endothelium, Descemet’s membrane and a thin, partial layer of the donor tissue’s stroma.

### Descemet’s membrane endothelial keratoplasty (DMEK)

DMEK involves the transplantation of only the Descemet’s membrane and endothelial layer of the cornea. DMEK has been described as a more technically challenging surgical procedure than DSAEK but also has been reported to provide better, post-transplant patient visual acuity, lower rejection rates and faster visual recovery.

### Deep anterior lamellar keratoplasty (DALK or ALK)

Is a partial thickness corneal transplant procedure used to treat disease or injury confined to anterior layers of the cornea: the epithelium, Bowman’s layer and stroma. DALK is most often used to treat keratoconus and corneal scarring.

### Distribution

A process that includes the receipt of a request for tissue, selection and inspection of the appropriate tissue and subsequent shipment and delivery of the tissue to the end user (surgeon) for utilization.

### Living donor

A donor where tissue is recovered from a live person; such as femoral heads which are recovered during total hip replacements or amnion which is recovered from the placenta in live births.

### Ocular

A general term which refers to the tissues of the eye which include the cornea and the sclera.

### Referral

A referral is when a deceased individual is referred to a donation organization or tissue bank for a determination of donation potential. In some jurisdictions all deaths are referred and in others frontline health professionals may do a pre-screening and only refer deaths which have no obvious contraindications to donation. A pre-screening which is reported to the tissue bank as a deferral is considered a referral as it has been evaluated for donor suitability.

### Released to inventory

Refers to grafts that has been evaluated, and deemed safe and suitable for transplantation, by a medical director, through the appropriate quality review and made available for transplantation. Prior to release grafts in the production process are considered quarantined.

### Sclera

The sclera is the part of the eye commonly known as the “white”. It forms the supporting wall of the eyeball, and is continuous with the clear cornea. Scleral grafts are widely used in ophthalmologic surgery.

### Soft tissue

A generic term for muscle, fat, fibrous tissue or other supporting tissue matrix. In tissue banking it often refers to fascia lata; the sheets of fibrous tissue enveloping, separating or binding together muscles and organs. Fascia lata is processed into grafts for use in surgical repairs.

### Structural bone grafts

These are bone grafts that are intended to support weight. They are classified into large or small. Large grafts include femurs, fibulas and humerus. Small grafts include sized grafts such as cortical dowels, wedges and rings.

### Surgical bone

Femoral heads can be recovered from total hip replacements and evaluated for suitability to transplant. These femoral heads are referred to as surgical bone. Surgeons grind the femoral head in the operating room to produce cancellous powder or particles. With the advent of bank produced pre-packaged cancellous and the increasing regulatory requirements the demand for surgical bone has declined.

### Tendon

Is a band of tough, inelastic fibrous tissue that connects a muscle with its boney attachment. Tendons commonly banked for use in sports medicine surgery include Achilles, Patellar and Tibialis.

### Tissue

Tissue is a general term which refers to non-ocular tissue; specifically musculoskeletal (bone), cardiac, skin and amnion tissue.

### Yield

Yield refers to the number of grafts which are recovered and released (deemed suitable) for transplant per donor. Yield can be affected by contamination, recovery technique, processing technique and donor factors such as age and comorbid diseases.

## Appendix C: List of contributing programs

British Columbia

- Eye Bank of British Columbia, Vancouver
- Island Health Bone Bank, Victoria

Alberta

- Southern Alberta Tissue Program, Calgary
- Lions Eye Bank of Calgary, Calgary
- Comprehensive Tissue Centre, Edmonton

Saskatchewan

- Saskatchewan Transplant Program, Saskatoon

Manitoba

- Tissue Bank Manitoba, Winnipeg
- Misericordia Eye Bank, Winnipeg

Ontario

Trillium Gift of Life Network manages the collation and submission of data from Ontario eye and tissue banks including:

- Eye Bank of Canada (Ontario Division), Toronto, Ontario
- The Hospital for Sick Children Tissue Laboratory, Toronto, Ontario
- Ontario Professional Fire Fighters Skin Bank, Toronto, Ontario
- Mount Sinai Allograft Technologies, Toronto, Ontario
- Lake Superior Centre for Regenerative Medicine, Thunder Bay, Ontario

Quebec

- Héma-Québec, Saint Laurent

New Brunswick

- New Brunswick Organ and Tissue Program; Saint John and Moncton

Nova Scotia

- Regional Tissue Bank, Halifax

## Appendix D: List of products programs distribute

**Table:**

Canadian Eye Banks	PK Corneas	DSAEK Corneas	DMEAK Corneas	Sclera	Amnion
Eye Bank of British Columbia, BC	Y	Y	*N	Y	N
Lions Eye Bank of Calgary, AB	Y	Y	N	Y	N
Comprehensive Tissue Centre, AB	Y	N	N	Y	Y
Saskatchewan Transplant	Y	N	N	Y	Y
Misericordia Eye Bank, MB	Y	N	*N	Y	Y
Eye Bank of Ontario, ON	Y	Y	*N	Y	Y
Héma-Québec, QU	Y	N	Y	Y	N
New Brunswick Organ and Tissue Program, NB	Y	N	N	Y	N
Regional Tissue Bank, NS	Y	Y	Y	Y	N

**Table:**

Canadian Tissue Banks	Cancellous bone	Structural bone	Rib or cartilage	Tendon	Fresh Osteo	Soft tissue	Cardiac	Skin
Island Health Bone Bank, BC (Surgical Bone	Y	N	N	N	N	N	N	N
Southern Alberta Tissue Program. AB	Y	Y	Y	Y	Y	Y	N	Y
Comprehensive Tissue Centre, AB	Y	Y	Y	Y	N	Y	Y	Y
Saskatchewan Transplant, SK	Y	Y	N	Y	N	N	N	N
Tissue Bank Manitoba	Y	Y	Y	Y	N**	Y	Y	Y
RegenMed, ON	Y	Y	N	Y	N	N	N	N
Mount Sinai Allograft Technologies, ON	Y	Y	N	Y	Y	N	N	N
Hospital for Sick Children Tissue, ON	N	N	N	N	N	N	Y	N
Ontario Professional Firefighters Skin Bank, ON	N	N	N	N	N	N	N	Y
Héma-Québec, QU	Y	Y	N	Y	N	N	Y	Y
New Brunswick Organ And Tissue, NB	Y	Y	N	N	N	N	N	N
Regional Tissue Bank,NS	Y	Y	N	Y	Y	Y	Y	Y

* DMEK processing in development for 2017

** Planning in place for fresh osteoarticular
